# Learning Response-Consistent and Background-Suppressed Correlation Filters for Real-Time UAV Tracking

**DOI:** 10.3390/s23062980

**Published:** 2023-03-09

**Authors:** Hong Zhang, Yan Li, Hanyang Liu, Ding Yuan, Yifan Yang

**Affiliations:** 1 School of Astronautics, Beihang University, Beijing 100191, China; 2 Institute of Artificial Intelligence, Beihang University, Beijing 100191, China

**Keywords:** unmanned aerial vehicle, discriminative correlation filter, response-consistent, background-suppressed, UAV tracking

## Abstract

With the advantages of discriminative correlation filter (DCF) in tracking accuracy and computational efficiency, the DCF-based methods have been widely used in the field of unmanned aerial vehicles (UAV) for target tracking. However, UAV tracking inevitably encounters various challenging scenarios, such as background clutter, similar target, partial/full occlusion, fast motion, etc. These challenges generally lead to multi-peak interferences in the response map that cause the target drift or even loss. To tackle this problem, a response-consistent and background-suppressed correlation filter is proposed for UAV tracking. First, a response-consistent module is developed, in which two response maps are generated by the filter and the features extracted from adjacent frames. Then, these two responses are kept to be consistent with the response from the previous frame. By utilizing the *l*2-norm constraint for consistency, this module not only can avoid sudden changes of the target response caused by background interferences but also enables the learned filter to preserve the discriminative ability of the previous filter. Second, a novel background-suppressed module is proposed, which makes the learned filter to be more aware of background information by using an attention mask matrix. With the introduction of this module into the DCF framework, the proposed method can further suppress the response interferences of distractors in the background. Finally, extensive comparative experiments have been conducted on three challenging UAV benchmarks, including UAV123@10fps, DTB70 and UAVDT. Experimental results have proved that our tracker has better tracking performance compared with 22 other state-of-the-art trackers. Moreover, our proposed tracker can run at ∼36 FPS on a single CPU for real-time UAV tracking.

## 1. Introduction

Target tracking is a research hotspot in machine vision and has been widely used in video surveillance, security, inspection, human–computer interaction, etc. In recent years, with the rapid development of unmanned aerial vehicles (UAV), target tracking has become one of its basic functions, which has been applied in aerial photogrammetry [1], autonomous landing [2], target location [3], aerial surveillance [4], object detection [5], etc. However, target tracking in UAVs has still encountered many challenges, such as background clutter, similar object, partial/full occlusion, and fast motion. Besides, most UAV platforms are based on CPUs with limited power supply and computing resources. The CPU-friendly tracker with excellent tracking performance and high FPS (Frames Per Second) is the research focus of UAV tracking. Since the speed requirement of real-time UAV tracking needs to exceed 30 FPS, improving the tracking speed (i.e., FPS) of trackers is particularly significant. Therefore, tracking methods with low complexity and high tracking accuracy in UAVs need to be studied.

Discriminative correlation filter (DCF) based methods have attracted extensive attention in UAVs because of their high computational efficiency and high tracking performance. These approaches learn a correlation filter by minimizing a ridge regression model, that is, minimizing the squared error of the actual target response and the ideal response. The actual target response is generated by a correlation operation between the filter and the features extracted from the search area. The ideal response is constructed as a 2-dimension (2D) ideal Gaussian distribution. Then, the location of the maximum actual response value is taken as the best target’s position. In addition, the DCF-based methods use the fast Fourier transform to convert the correlation operation in the spatial domain into the dot-product operation in the frequency domain, which significantly reduces the computational complexity and is suitable for the CPUs platform on UAV. By means of the cyclic operation in the Fourier domain, the DCF-based methods also generate many implicit training samples. However, there are still some problems with the DCF-based methods to be solved for target tracking.

First of all, the cyclic shift operation of DCF-based methods in the Fourier domain will lead to training samples discontinuity at the boundary, namely the boundary effect that reduces the reliability of the samples and degrades the tracking performance. To remedy this drawback, some methods [6,7,8,9,10] have developed the spatial regularization term to alleviate the boundary effect, which makes the filter pay more attention to the information of the target area and penalizes the filter coefficients far from the target region. Some approaches [11,12] expand the search area and crop the real positive and negative samples from the search area. Secondly, since the ideal response used to train the filter presents a 2D Gaussian distribution, the actual response generated by the learned filter should also exhibit a similar distribution, i.e., a sharp peak with a maximum value in the target region and low values tending to zero in the background area. Unfortunately, there still exist distractors in various scenarios during the tracking process. As a result, the distribution of the actual response will not be an ideal Gaussian shape. In others words, the actual distribution contains multi-peaks that significantly affect the target location. To suppress the multi-peak response interferences, some methods [13,14,15] adopt the response regularization to smooth response variations and repress the abnormal response peaks appearing in the current frame. Some methods [16,17] introduce the context information into the DCF framework for training the filter, which effectively reduces the response fluctuations of distractors.

**Figure 1:**
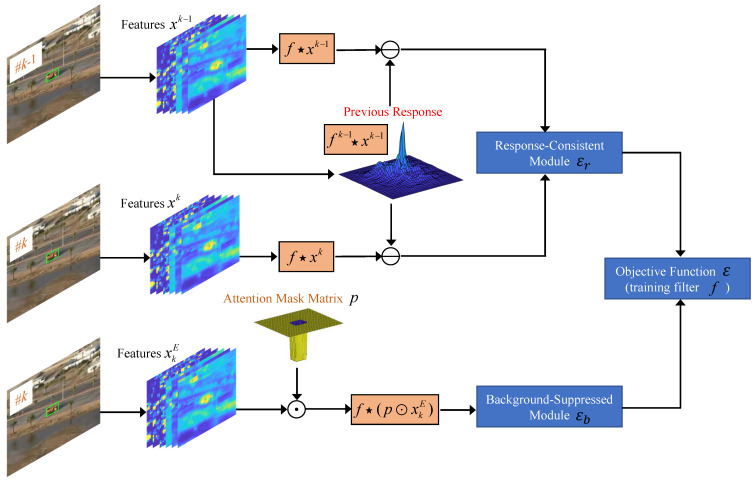
Overall tracking framework of our tracker. The previous response $\left(f^{k-1}⋆x^{k-1}\right)$ is used as a consistency reference for constraining two responses, that is, $\left(f⋆x^{k-1}\right)$ and $\left(f⋆x^{k}\right)$. Then, the differences, i.e., $\left(f⋆x^{k}-f^{k-1}⋆x^{k-1}\right)$ and $\left(f⋆x^{k-1}-f^{k-1}⋆x^{k-1}\right)$, are both introduced into the response-consistent module $E_{r}$. In addition, the attention mask matrix *p* is employed to improve the filter’s perception of background information and incorporated into the background-suppressed module $E_{b}$ for suppressing the distractors around the target. Finally, both response-consistent and background-suppressed modules are integrated into the objective function. ⋆ represents the operation of circular correlation and ⊙ is the Hadamard product. ⊖ denotes the subtraction operation. $x^{k}$ stems from the appearance model in Section 3.6. $x_{k}^{E}$ represents the extracted features in *k*-th frame.

In this paper, we propose a response-consistent and background-suppressed correlation filter for UAV tracking, which utilizes the SRDCF [6] as the baseline tracker. The overall tracking framework is shown in Figure 1. Specifically, a novel response-consistent module is developed, in which two response maps are generated by the filter and the features extracted from the current frame and the previous frame, respectively. Then, this module uses the *l*2-norm constraint to keep the two responses consistent with the known response from the previous frame. With the constraint operation, this module enables the proposed tracker not only to avoid sudden changes in the target response caused by background interferences but also to preserve the filter’s discriminative ability inherited from the previous filter. Besides, a novel background-suppressed module is proposed, which enables the filter to focus on the background region by using an attention mask matrix. Relying on this module, the filter can learn the additional background information and prevent the influence of distractors in the background. Furthermore, both above modules are simultaneously introduced into the DCF framework and solved by the ADMM method [18]. Finally, we have conducted a comprehensive evaluation of our tracker on UAV123@10fps [19], DTB70 [20] and UAVDT [21] benchmarks. Compared with 22 other state-of-the-art trackers, our tracker achieves significant tracking performance with handcrafted features and runs at ∼36 frames per second (FPS) on a single CPU for real-time UAV tracking.

The contributions in this paper are summarized as follows:

1. A novel response-consistent module is proposed to minimize the difference between the responses obtained by the filter in adjacent frames, which avoids mutations of the target response caused by background interferences, and thus helps to enhance the discriminative ability of the learned filter.
2. A novel background-suppressed module is proposed, which employs an attention mask matrix to make the learned filter identify the background information, and thus represses the interferences caused by distractors.
3. Experimental results on three public challenging UAV benchmarks have been completed and demonstrate that our tracker achieves excellent tracking performance against 22 other state-of-the-art trackers. Our tracker can realize real-time tracking on a single CPU platform.

## 2. Related Work

In this section, we mainly discuss the object tracking methods related to our work in this paper, i.e., the discriminative correlation filter methods.

### 2.1. Discriminative Correlation Filter Methods

Considering that DCF-based methods have the advantages of high computation efficiency, high tracking accuracy and convenient deployment on CPUs devices, they have attracted attention in UAVs. Bolme et al. [22] introduced the correlation filter method into the object tracking field for the first time. Typically, they transferred the correlation operation in the spatial domain to the dot-product operation in the frequency domain, which greatly reduces the computational complexity. Henriques et al. [23] introduced the cyclic shift and fast Fourier transform into the DCF framework. In [23], a dense sampling method based on the cyclic shift operation is utilized to increase the training samples. Then, Henriques et al. [24] proposed a kernelized correlation filter (KCF). KCF has used circulant matrices to generate many training samples and improved the tracking robustness by using the multi-channel HOG [25] feature instead of the single-channel pixel feature in [23]. However, the HOG feature can describe the appearance and shape of the tracked object, which is unable to express the color attribute. Therefore, Martin et al. [26] proposed an adaptive color attribute method for object tracking, which uses the Principle Component Analysis (PCA) method to reduce the feature dimensions. Li et al. [27] proposed a scale-adaptive kernel correlation filter (SAMF), which fuses the HOG and CN [28] features and employs the scale pyramid method to find the optimal scale size of the target. Bertinetto et al. [29] proposed the Staple tracker, which locates the target by fusing the template response and histogram response generated by the HOG feature and color histogram, respectively.

In order to improve the tracking performance of DCF-based methods, several methods equipped with deep features have been proposed in recent years. Martin et al. [30] proposed the C-COT tracker by learning continuous convolution operators with deep multi-resolution features. To accelerate the tracking speed of C-COT, Martin et al. [31] exploited the ECO tracker, which makes use of factorized convolution operator and generative sample model for reducing complexity. In [9], Dai et al. proposed an adaptive spatially-regularized correlation filter (ASRCF). The ASRCF tracker adopts an automatically estimated target-aware spatial regularization to flexibly generate filter penalty coefficients and uses the combination of shallow and deep features for target location. In [32], Xu et al. proposed the GFS-DCF tracker, which uses the group feature selection method to select reliable channel features among the handcrafted and deep CNN features. In addition, GFS-DCF employs a temporal smoothness regularization term and a low-rank approximation method for training correlation filter.

To sum up, some of the above-mentioned methods use the cyclic shift to construct training samples, which generally leads to the existence of the unwanted boundary effect that degrades the reliability of training samples. Some methods usually make use of the combined features to improve the tracking performance and utilize spatial or temporal regularization to constrain the filter. Different from them, the proposed method here takes into account the consistency of the response generated by the filter and the features in adjacent frames. Moreover, in order to increase the filter’s ability to perceive background information, a new background-suppressed module is introduced into the DCF framework for repressing the interferences of distractors.

### 2.2. Tracking with Reducing Boundary Effect

In order to alleviate the boundary effect, several related pieces of research have been studied. Martin et al. [6] proposed a spatially regularized correlation filter (SRDCF). SRDCF makes use of a spatial penalty term to prevent the filter from learning the information of the regions far from the target so that the boundary of training samples can be repressed. Lukezic et al. [7] proposed the CSRDCF tracker, which employs the foreground/background histogram to automatically estimate a spatial reliable map. By restricting this spatial map to be suitable for the irregular shape of the target, CSRDCF can expand the search range for mitigating the boundary effect and improve the tracking performance of non-rectangular objects. Galoogahi et al. [12] developed a background-aware correlation filter (BACF) with the HOG feature. In BACF, the target search region is expanded and the real positive and negative training samples are cropped by using a clipping matrix. With these cropped samples, BACF can avoid generating training samples by the cyclic shift and improve the authenticity of samples. Li et al. [8] proposed a spatial-temporal regularized correlation filter (STRCF) based on SRDCF, which makes the learned filter consistent with the historical filter against the filter’s sudden change. In addition, STRCF has adopted the alternating direction method of multipliers (ADMM) method [18] to solve sub-problems in the frequency domain. Compared with the gauss-seidel method of SRDCF, the computational efficiency of STRCF has been greatly improved. Li et al. [10] proposed an automatic spatial-temporal regularized correlation filter (AutoTrack). In [10], local and global response variations are employed to automatically adjust spatial and temporal regularization parameters for facing different tracking scenarios. Besides, the ADMM method is also used to solve the sub-problems in AutoTrack.

### 2.3. Tracking with Response-Based Approach

Lin et al. [33] proposed a method with the response-based bidirectional incongruity by using the inter-frame temporary block information. In [33], a novel bidirectional incongruity regularization is exploited, which aims to make the learned filter still have the discriminative ability of previous filters and enhance the generalization capability against appearance changes. Later, Lin et al. [34] proposed an adaptive response reasoning approach for UAV tracking. In [34], the historical response regularization produces a training label generated by the features and the filter from the previous frame. This label is used as a regression term for training the current filter with the previous features. In addition, [34], constructs the inferred response regularization by means of the prediction ability of the previous filter, which aims to repress the violent response fluctuations.

Compared with [33,34], the proposed method is different from them. The method in our work focuses on keeping the current response consistent with the previous response to avoid sudden response changes by using the l2-norm constraint term, i.e., $\left(f⋆x^{k}-f^{k-1}⋆x^{k-1}\right)$. However, the methods in [33,34] center on constraining the error of the response generated by the current and historical filters on the same feature and do not use the previous response as a consistency reference. Furthermore, considering the impact of distractors in the background during the tracking process, we also have introduced an independent background-suppressed module into the training model, which uses an attention matrix to make the filter focus on learning the features extracted from the background. With the background-suppressed module, the method proposed in our work is also different from [33,34].

### 2.4. Tracking with Background Distractors Suppression

Suppressing the distractors in the background is a challenging problem in the field of object tracking. Recently, several DCF-based methods have been developed. Mueller et al. [16] exploited a context-aware correlation filter (CACF) by introducing the contextual information around the target into the filter training model. The CACF tracker can effectively suppress the responses of background patches and improve the discrimination ability of the filter. Zhang et al. [17] proposed a sparse learning-based correlation filter (SRCF), which adds a sparse response regularization term in the DCF framework and trains the filter in a ridge regression model. With the experimental verification, the SRCF tracker can reduce the abnormal responses in the background region and remove the unexpected peaks caused by distractors. Fu et al. [35] proposed a dynamic regression method (DR2Track) for automatically repressing the distractors around the target. In [35], DR2Track has replaced the ideal Gaussian label with a dynamic regression label generated by the local maximum values of the response during the detection stage. Therefore, DR2Track can automatically detect potential distractors and repress the response peaks belonging to them in the background. Huang et al. [13] developed an aberrant repressed correlation filter (ARCF), which uses the shift operation to coincide the current response with the previous response. After keeping the two responses consistent by dint of the *l*2-norm constraint, ARCF can achieve the response aberrance repressed and ensure the target is tracked stably in the environment of background clutter. Zhang et al. [14] proposed a tracking method with dual regression for target awareness and background suppression (TABSCF), which adopts the saliency detection method to extract the target features. Moreover, TABSCF uses target features and extra global features to regress the target filter and the global filter, respectively. Finally, by restricting two responses generated by the two regressed filters, TABSCF can acquire a superior ability to suppress the background distractors. Compared with the above methods, we adopt an independent regularization in which an attention matrix applies high weights on the background area to train the filter for attaining low response on distractors.

Huang et al. [36] proposed a multi-channel background suppressed correlation filter (BSCF) for target tracking. The BSCF has constructed a training sample that contains all the background, in which the target area is padded with zeros. Then, this sample needs to be extracted features again for training the filter. Liu et al. [37] proposed a novel tracker with a masked correlation filter, which masks the training samples with a rectangle to preserve the target area and remove the background area for decreasing noise in the background. Wang et al. [38] proposed a DCF-based method that utilizes a steeply decreasing mask matrix to repress the information in the background and integrate the mask matrix into the regression term with a Gaussian label. Huo et al. [39] proposed a soft mask correlation filter, which exploits a soft mask to pay attention to the target patch and crop the area around the target by applying a mask value of zero. Compared with [36], the background-suppressed module in our work obtains the sample directly in the current frame without padding zeros in the target area. An attention matrix is used to highlight the background area and weaken the target area of features. Besides, since we do not destroy the sample structure, the sample can directly participate in target appearance model updating. However, in BSCF, the target appearance model and background sample need to be updated separately. Compared with [37,38,39], our method does not choose the way that removes the background information in [37], sets the pixels of the background away from the target to zero and crops the target area for training model in [38,39]. By contrast, we apply low and high weights on the area of the target and background with the proposed matrix for learning the filter. Furthermore, the attention matrix exists in an independent regularization and does not act in the regression term with a Gaussian label.

## 3. Proposed Method

### 3.1. Baseline Tracker

Given the training sample set $X^{\left(k\right)}=\left[x_{1}^{\left(k\right)},x_{2}^{\left(k\right)},x_{3}^{\left(k\right)},\ldots ,x_{D}^{\left(k\right)}\right]$ with the extracted features $x_{d}^{\left(k\right)}\in R^{N}$(d=1,2,3…D) in the *k*-th frame and the ideal Gaussian response label $y\in R^{N}$, the overall objective function of SRDCF [6] is to optimize the ridge regression problem as follows:

(1) $$E\left(f\right)=\sum_{k=1}^{N}\alpha^{\left(k\right)}∥y_{d}-\sum_{d=1}^{D}f_{d}⋆x_{d}^{\left(k\right)}{∥}_{2}^{2}+\sum_{d=1}^{D}{∥w⊙f_{d}∥}_{2}^{2},$$

where ⋆ denotes the operation of circular correlation, *D* is the number of feature channels, ⊙ stands for the Hadamard product, ${∥.∥}_{2}^{2}$ represents the sum of squares, $\alpha^{\left(k\right)}$ is a weight coefficient, *f* represents the filter to be learned in the *k*-th frame and $f=[f_{1},f_{2},f_{3},\ldots ,f_{d}]$, $f_{d}\in R^{N}$(d=1,2,3,…,D), and *w* is the spatial regularization penalty weight, which is assigned higher coefficients in the background region than that in the target region. Owing to the usage of *w* in the objective function, the learned filter will focus on the reliable target region and produce a low response on background patches.

As a DCF-based tracker, SRDCF can achieve stable tracking in most normal scenarios by alleviating the boundary effect and expanding the search area. However, complex background interferences usually occur in UAV tracking and cause the target drift or loss when the response values of distractors exceed the response value located at the target region. In view of this problem, considering that SRDCF has not used the feature information of adjacent frames, it’s easy to locate the target incorrectly when distractors exist in the search area. Moreover, SRDCF makes the filter focus on the target area by using the spatial penalty term and fails to effectively learn the background information, which leads to certain deficiencies in the filter’s ability to suppress background interferences.

### 3.2. Response-Consistent Module

The tracked target often encounters various scenarios in UAV tracking, such as background clutter, similar object, occlusion and fast motion, etc. Therefore, the response, which is generated by the filter and the features extracted from the search region, usually contains multi-peaks that represent the possibility of becoming a target in the corresponding position. Supposing that the maximum value of a certain interference peak is larger than the target’s, the target will be positioned incorrectly. To efficiently repress the multi-peak response interferences, the response-consistent module is introduced, which aims to maintain the response consistency in adjacent frames and constrain the response variations to prevent the occurrence of distractor peaks. The formulation is as follows:

(2) $$E_{r}=\gamma_{1}\sum_{d=1}^{D}∥f_{d}⋆x_{d}^{\left(k\right)}-f_{d}^{\left(k-1\right)}⋆x_{d}^{\left(k-1\right)}{∥}_{2}^{2}+\gamma_{2}\sum_{d=1}^{D}∥f_{d}⋆x_{d}^{\left(k-1\right)}-f_{d}^{\left(k-1\right)}⋆x_{d}^{\left(k-1\right)}{∥}_{2}^{2},$$

where $\gamma_{1}$ and $\gamma_{2}$ are two fixed regularization parameters, $x_{d}^{\left(k\right)}$ equals the appearance model $x_{model}^{\left(k\right)}$ in Section 3.6 that has been updated before solving the filter $f_{d}$. In Equation (2), the $E_{r}$ can be divided into two parts, $E_{r1}=\gamma_{1}\sum_{d=1}^{D}∥f_{d}⋆x_{d}^{\left(k\right)}-f_{d}^{\left(k-1\right)}⋆x_{d}^{\left(k-1\right)}{∥}_{2}^{2}$ and $E_{r2}=\gamma_{2}\sum_{d=1}^{D}∥f_{d}⋆x_{d}^{\left(k-1\right)}-f_{d}^{\left(k-1\right)}⋆x_{d}^{\left(k-1\right)}{∥}_{2}^{2}$. The $E_{r1}$ is constructed to keep the current and previous responses consistent, which can prevent the current response from abruptly changing due to the sudden change of the features occurring in the current frame. Then, the $E_{r2}$ is employed to make the response generated by $f=[f_{1},f_{2},\ldots ,f_{D}]$ and $X^{\left(k-1\right)}=\left[x_{1}^{\left(k-1\right)},x_{2}^{\left(k-1\right)},\ldots ,x_{D}^{\left(k-1\right)}\right]$ and the previous response constrain each other. Therefore, the filter *f* can inherit the discriminative ability of the previous filter $f^{\left(k-1\right)}$. By employing $E_{r1}$ and $E_{r2}$ jointly, both the response and the filter in the current *k*-th frame will maintain historical continuity, respectively. Besides, since the current filter *f* is trained based on the two features that are $X^{\left(k\right)}$ and $X^{\left(k-1\right)}$, the filter *f* is less sensitive to the distractors that are suddenly present in the search area of the *k*-th frame, which enables the target to be stably tracked.

### 3.3. Background-Suppressed Module

In Section 3.2, although the response-consistent module can alleviate the influence of distractors that appeared in the search area, it cannot learn more background information. To enable the filter *f* to learn the background information and further suppress the interferences in the background, the background-suppressed module is introduced, which uses an attention mask matrix *p* to make the filter *f* focus on the background region. The formulation can be seen as follows:

(3) $$E_{b}=\gamma_{3}\sum_{d=1}^{D}{∥f_{d}⋆\left(p⊙x_{d}^{E}\right)∥}_{2}^{2},$$

where $\gamma_{3}$ is a fixed regularization parameter. In Equation (3), *p* is an attention mask matrix, in which the values belonging to the target region are lower than the background region, and $X^{E}=\left[x_{1}^{E},x_{2}^{E},x_{3}^{E},\ldots ,x_{D}^{E}\right]$(d=1,2,3,…,D) represents the features that are extracted in the current *k*-th frame. In this module, a Hadamard product operation between *p* and $X^{E}$ is conducted, which increases the weight of background information. Then, a correlation operation with the Hadamard product result and the filter *f* is performed. Finally, the constraint of l2-norm is used for facilitating the minimization of the ridge regression model.

### 3.4. Overall Objective Function

Based on SRDCF [6], the overall objective function that has been proposed here can be constructed as follows:

(4) $$E\left(f\right)=\sum_{d=1}^{D}∥y_{d}-f_{d}⋆x_{d}^{\left(k\right)}{∥}_{2}^{2}+\sum_{d=1}^{D}{∥w⊙f_{d}∥}_{2}^{2}+E_{r}+E_{b},$$

where $E_{r}$ is the response-consistent module, and $E_{b}$ is the background-suppressed module. Considering that every regularization term in Equation (4) contains *D* times of independent computation where *D* is the number of feature channels, we can decompose Equation (4) into *D* subproblems and choose the *d*-th channel to derive. In order to simplify the following derivation process, we omit the subscript ${\left(\cdot \right)}_{d}$. Therefore, the *d*-th subproblem for derivation can be formulated as follows:

(5) $$\begin{array}{cc} arg\underset{f}{min}\; & ∥y-f⋆x^{\left(k\right)}{∥}_{2}^{2}{+∥w⊙f∥}_{2}^{2}+\gamma_{1}{∥f⋆x^{\left(k\right)}-f^{\left(k-1\right)}⋆x^{\left(k-1\right)}∥}_{2}^{2}\\ \; & +\gamma_{2}∥f⋆x^{\left(k-1\right)}-f^{\left(k-1\right)}⋆x^{\left(k-1\right)}{∥}_{2}^{2}+\gamma_{3}{∥f⋆\left(p⊙x^{E}\right)∥}_{2}^{2}. \end{array}$$

### 3.5. Optimization

By introducing an auxiliary variable $h\in R^{N}$ and taking h=f, we can make Equation (5) be an equality constraint formulation as follows:

(6) $$\begin{array}{ll} arg\underset{f}{min}\; & ∥y-f⋆x^{\left(k\right)}{∥}_{2}^{2}{+∥w⊙f∥}_{2}^{2}+\gamma_{1}{∥f⋆x^{\left(k\right)}-f^{\left(k-1\right)}⋆x^{\left(k-1\right)}∥}_{2}^{2}\\ & +\gamma_{2}∥f⋆x^{\left(k-1\right)}-f^{\left(k-1\right)}⋆x^{\left(k-1\right)}{∥}_{2}^{2}+\gamma_{3}{∥f⋆\left(p⊙x^{E}\right)∥}_{2}^{2},\\ s.t.\;\;\;\;\; & \;h=f. \end{array}$$

In Equation (6), there is the correlation operation in the spatial domain, which has a certain high complexity. Therefore, to promote computational efficiency, the correlation operation is converted to the element-wise operation in the frequency domain. With the Parseval’s theorem, Equation (6) can be formulated as follows:

(7) $$\begin{array}{ll} arg\underset{\hat{f}}{min}\; & \{∥\hat{y}-{\hat{f}}^{*}⊙{\hat{x}}^{\left(k\right)}{∥}_{2}^{2}{+∥w⊙f∥}_{2}^{2}+\gamma_{1}∥{\hat{f}}^{*}⊙{\hat{x}}^{\left(k\right)}-{\hat{f}}^{*(k-1)}⊙{\hat{x}}^{\left(k-1\right)}{∥}_{2}^{2}\\ & +\gamma_{2}∥{\hat{f}}^{*}⊙{\hat{x}}^{\left(k-1\right)}-{\hat{f}}^{*(k-1)}⊙{\hat{x}}^{\left(k-1\right)}{∥}_{2}^{2}+\gamma_{3}∥{\hat{f}}^{*}⊙\hat{x^{E^{\prime }}}{∥}_{2}^{2}\},\\ s.t.\;\;\;\;\; & \;\hat{f}-\sqrt{N}Fh=0, \end{array}$$

where the subscript  $\hat{\;}$  indicates the discrete Fourier transformation (DFT) and the subscript * represents the complex conjugation. In Equation (7), $F\in C^{N\times N}$ is the DFT matrix that can convert the vector *k* in the spatial domain into the vector $\hat{k}$ in the frequency domain, i.e., $\hat{k}=\sqrt{N}Fk$. For simplification, the Lagrange multiplier $\hat{\zeta }\in C^{N}$ is adopted to convert Equation (7) into the augmented Lagrangian form for facilitating derivation with the ADMM method. Then, Equation (7) can be reformulated as follows:

(8) $$\begin{array}{ll} L(\hat{f},\hat{\zeta }) & =∥\hat{y}-{\hat{f}}^{*}⊙{\hat{x}}^{\left(k\right)}{∥}_{2}^{2}{+∥w⊙f∥}_{2}^{2}+\gamma_{1}∥{\hat{f}}^{*}⊙{\hat{x}}^{\left(k\right)}-{\hat{f}}^{*(k-1)}⊙{\hat{x}}^{\left(k-1\right)}{∥}_{2}^{2}\\ & +\gamma_{2}∥{\hat{f}}^{*}⊙{\hat{x}}^{\left(k-1\right)}-{\hat{f}}^{*(k-1)}⊙{\hat{x}}^{\left(k-1\right)}{∥}_{2}^{2}+\gamma_{3}∥{\hat{f}}^{*}⊙\hat{x^{E^{\prime }}}{∥}_{2}^{2}\\ & +\mu ∥\hat{f}-\sqrt{N}Fh+\frac{1}{\mu }\hat{\zeta }{∥}_{2}^{2}, \end{array}$$

where μ represents a penalty factor, $x^{E^{\prime }}$ is equal to $p⊙x^{E}$.

With the ADMM method and the augmented Lagrangian form of Equation (8), we decompose Equation (8) into two subproblems for derivation, respectively. Each subproblem has a closed-form solution.

Subproblem $\hat{f}$:

Solving for the solution of $\hat{f}$, the independent auxiliary variable *h* and $\hat{\zeta }$ can be fixed and be introduced into the ADMM iteration process for derivation. Therefore, the optimized derivation result ${\hat{f}}^{\left(i+1\right)}$ can be formulated as follows:

(9) $$\begin{array}{ll} {\hat{f}}^{\left(i+1\right)} & =arg\underset{\hat{f}}{min}\;\left\{∥\hat{y}-{\hat{f}}^{*}⊙{\hat{x}}^{\left(k\right)}{∥}_{2}^{2}+\gamma_{1}∥{\hat{f}}^{*}⊙{\hat{x}}^{\left(k\right)}-{\hat{f}}^{*(k-1)}⊙{\hat{x}}^{\left(k-1\right)}{∥}_{2}^{2}\right.\\ & +\gamma_{2}∥{\hat{f}}^{*}⊙{\hat{x}}^{\left(k-1\right)}-{\hat{f}}^{*(k-1)}⊙{\hat{x}}^{\left(k-1\right)}{∥}_{2}^{2}+\gamma_{3}∥{\hat{f}}^{*}⊙\hat{x^{E^{\prime }}}{∥}_{2}^{2}\\ & \left.+\mu ∥\hat{f}-\sqrt{N}Fh+\frac{1}{\mu }\hat{\zeta }{∥}_{2}^{2}\right\}, \end{array}$$

where *i* represents the *i*-th iteration. In order to obtain the solution of Equation (9), the minimization result of Equation (9) can be considered zero. Therefore, with the simplification, ${\hat{f}}^{\left(i+1\right)}$ is obtained as follows:

(10) $$\begin{array}{c} {\hat{f}}^{\left(i+1\right)}=\frac{{\hat{x}}^{\left(k\right)}⊙{\hat{y}}^{*}+\left(\gamma_{1}{\hat{x}}^{\left(k-1\right)}⊙{\hat{x}}^{*(k-1)}+\gamma_{2}{\hat{x}}^{\left(k-1\right)}⊙{\hat{x}}^{*(k)}\right)⊙{\hat{f}}^{\left(k-1\right)}+\mu \sqrt{N}Fh^{i}-{\hat{\zeta }}^{\left(i\right)}}{\left(1+\gamma_{2}\right)\left({\hat{x}}^{\left(k\right)}⊙{\hat{x}}^{*(k)}\right)+\gamma_{1}\left({\hat{x}}^{\left(k-1\right)}⊙{\hat{x}}^{*(k-1)}\right)+\gamma_{3}\left(\hat{x^{E^{\prime }}}⊙{\hat{x^{E^{\prime }}}}^{*}\right)+\mu }, \end{array}$$

where the fraction operation in Equation (10) is the element-wise division.

Subproblem *h*:

Solving for the solution of *h*, the independent auxiliary variable $\hat{f}$ and $\hat{\zeta }$ can be fixed and be introduced into the ADMM iteration process for derivation. Therefore, the optimized derivation result $h^{\left(i+1\right)}$ can be formulated as follows:

(11) $$\begin{array}{c} h^{\left(i+1\right)}=arg\underset{h}{min}\;\left\{∥w⊙h^{\left(i\right)}{∥}_{2}^{2}+\mu ∥\hat{f}-\sqrt{N}Fh^{\left(i\right)}+\frac{1}{\mu }{\hat{\zeta }}^{\left(i\right)}{∥}_{2}^{2}\right\}. \end{array}$$

In order to obtain the solution of Equation (11), we can regard the derivation of *h* as zero. Hence, a closed-form solution to $h^{\left(i+1\right)}$ can be seen as follows:

(12) $$\begin{array}{c} h^{\left(i+1\right)}=\frac{F^{-1}\left(\mu {\hat{f}}^{\left(i+1\right)}+{\hat{\zeta }}^{\left(i\right)}\right)}{\frac{1}{N}\left(w⊙w^{*}\right)+\mu }, \end{array}$$

where $F^{-1}$ denotes the inverse discrete Fourier transformation.

Updating Lagrangian Multiplier $\hat{\zeta }$:

The lagrangian multiplier $\hat{\zeta }$ is usually updated as follows:

(13) $$\begin{array}{c} {\hat{\zeta }}^{\left(i+1\right)}={\hat{\zeta }}^{\left(i\right)}+\mu \left({\hat{f}}^{\left(i+1\right)}-{\hat{h}}^{\left(i+1\right)}\right). \end{array}$$

Besides, $\mu^{\left(i+1\right)}$ is updated by $\mu^{\left(i+1\right)}=min\left(\beta \mu^{\left(i\right)},\mu_{max}\right)$ at the last step of the ADMM iterations. β is a fixed step scale factor and $\mu_{max}$ is a fixed maximum value of μ.

### 3.6. Updating Appearance Model

The method of updating the appearance model in this paper is consistent with most DCF-based methods. The updated way is as follows:

(14) $$\begin{array}{c} {\hat{x}}_{model}^{\left(k\right)}=\left(1-\eta \right){\hat{x}}_{model}^{\left(k-1\right)}+\eta \hat{x^{E}}, \end{array}$$

where ${\hat{x}}_{model}^{\left(k\right)}$ and ${\hat{x}}_{model}^{\left(k-1\right)}$ represent the appearance model in the *k*-th and (k−1)-th frames. η is the fixed online learning rate of the appearance model.

### 3.7. Target Localization and Scale Estimation

Target Localization. To obtain the target location in the *k*-th frame, we first get the search region of the target in the *k*-th frame by using the known information, i.e., the target position and scale from the (k−1)-th frame. Then, we use the filter $f^{\left(k-1\right)}$ of the (k−1)-th frame and the extracted features $x^{E}$ of the search region in the *k*-th frame to generate the response as follows:

(15) $$\begin{array}{c} R^{\left(k\right)}=F^{-1}\left({\hat{f}}^{\left(k-1\right)}⊙\hat{x^{E}}\right). \end{array}$$

The target in the *k*-th frame can be located by finding the position of the maximum value of the response $R^{\left(k\right)}$.

Scale Estimation. In order to obtain the scale of the target, we adopt the method following ARCF [13]. First, we use the learned filter in Equation (10) to calculate the responses, which belonged to target regions of 33 different scales, respectively. Then, we choose the scale, which corresponds to the response that has the maximum value, as the best scale of the target in the *k*-th frame.

### 3.8. Tracking Pipeline

The tracking pipeline of the proposed tracker RCBSCF is summarized in Algorithm 1. In the detection stage, RCBSCF first extracts the features from the currently input frame with the known position and scale predicted in the previous frame. Then, with the correlation operation between the current features and the filter of the previous frame, a response map can be generated. The target’s position is the location of the maximum value in the response map. In addition, by using the scale estimation method in Section 3.7, the best scale of the target is obtained. In the training stage, RCBSCF extracts the features in the current frame by using the position and scale that have been obtained in the detection stage. By means of Equation (14), the appearance model is updated. After that, the updated appearance model is brought into Equation (4) in Section 3.4 for learning the filter.
**Algorithm 1:** Response-Consistent and Background-Suppressed Correlation Filter (RCBSCF)
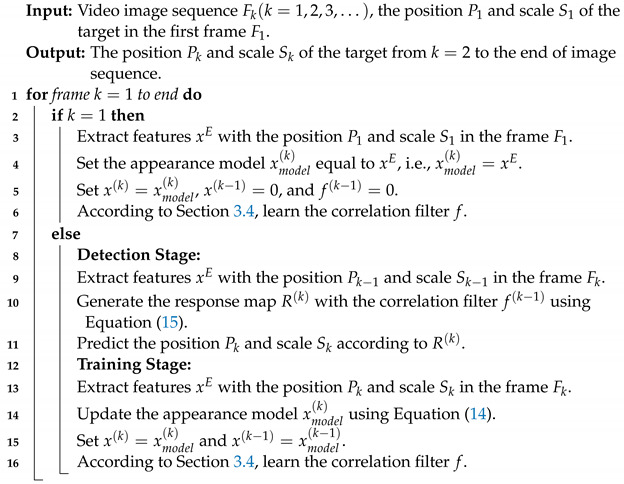


## 4. Experiments

In this section, we have conducted a comprehensive experimental analysis of the proposed tracker on three public UAV benchmarks, i.e., UAV123@10fps [19], DTB70 [20] and UAVDT [21]. Besides, we have compared our tracker with other two groups of a total of 22 state-of-the-art trackers, including 13 handcrafted features-based trackers (i.e., AutoTrack [10], ARCF [13], DR2Track [35], LADCF-HC [40], STRCF [8], MCCT-H [41], KCC [42], BACF [12], fDSST [43], SRDCF [6], Staple_CA [16], KCF [24], SAMF [27]) and 9 deep features-based trackers (i.e., ECO [31], SiamFC [44], IBCCF [45], UDT [46], UDT+ [46], TRACA [47], DSiam [48], LUDT [49], LUDT+ [49]).

### 4.1. Implementation Details

All the experiments are implemented on MATLAB R2019a with an Intel i5-9600K CPU, 32G RAM and a single NVIDIA GTX2070 GPU. The RCBSCF tracker uses the handcrafted features that contain HOG (31 channels), CN (10 channels) and gray-scale (1 channel) features for target tracking. We set the values of $\gamma_{1}$, $\gamma_{2}$ and $\gamma_{3}$ equal to 37, 16.4 and 6, respectively. As for the initial penalty factor μ and the maximum value $\mu_{max}$, we set them to 100 and $10^{5}$, respectively. The penalty scale step β is set to 500. The ADMM iterations are set to 5 and the learning rate η for updating the appearance model is 0.0327. We set the search region to be a square area, of which the side length is $\sqrt{5WH}$ (*W* and *H* are the width and height of the target). The feature cell size is set to 4. In addition, following the setting of STRCF, we set the values of *w* that are belonged to the target region and background region to $10^{-3}$ and $10^{5}$, respectively. With the same size of the target region and background region, we set the values of *p* that are belonged to the target region and background region to 0.1 and 1.2, respectively. The total size of *p* is equal to *w*.

**Remark** **1.** *The parameter μ is used to penalizes the term, i.e., $∥\hat{f}-\sqrt{N}Fh+\frac{1}{\mu }\hat{\zeta }{∥}_{2}^{2}$, in Equation (9). Generally, given that μ is large enough, the optimal solution of Equation (9) can be obtained, which approximates the solution of the rest of Equation (9) except for $∥\hat{f}-\sqrt{N}Fh+\frac{1}{\mu }\hat{\zeta }{∥}_{2}^{2}$. Here, the initial μ usually ranges from 1 to $\mu_{max}$, which determines the initial penalized degree. $\mu_{max}$ is empirically set to $10^{5}$ for limiting the maximum value of μ, which will retain the stability of the solved filter. β is a step scale factor and $1⩽\beta ⩽\frac{\mu_{max}}{\mu }$. Considering that the large values of μ and β will help the ADMM to converge [12], the initial μ and β are empirically set to 100 and 500, respectively. The number of ADMM iterations affects the optimal solution of the filter and the computing efficiency, which is usually not greater than 5. The learning rate η is used to update the appearance model and prevent the tracker from being affected by the severe change in target appearance. With 0<η⩽1, the updated appearance model is mainly composed of historical appearances. The square search region is generally set to be larger than the size of the target, which is convenient for capturing the target and generating a large number of positive and negative samples. The square search region is usually 2 to 5 times the size of the target. A feature cell is a unit for feature extraction, which affects the size of the feature map as well as the computation and tracking precision of the tracker. The feature cell size can be N × N (N = 2, 3, 4, …, 8). The parameter w aims to penalize the filter’s coefficients in the background of the search region and makes the filter focus on the target. In our work, the size of the square search region, the feature cell size and w are following the setting in [8]. The parameter p is a feature weight, which enlarges the feature values located at the background region for penalizing the background response generated by the filter. To ensure that the weighted feature has not changed greatly, the values of p in the background and target regions are set to close 1, which range from 1 to 2 and from 0 to 1, respectively.*

### 4.2. Benchmark and Metric

The proposed tracker in this paper is evaluated on three public challenge benchmarks, which are UAV123@10fps, DTB70 and UAVDT, respectively. These benchmarks contain a total of 243 video sequences and have over 90K pictures, which cover various UAV tracking scenarios.

Benchmark. The UAV123@10fps benchmark is a fully annotated UAV video image sequence, which contains 123 sequences. UAV123@10fps is generated by down-sampling from the UAV123 dataset, which increases the difficulty of object tracking over UAV123. UAV123@10fps has 103 high-resolution sequences of different objects captured by UAV camera at heights from 5 to 25 m, 12 low-quality and low-resolution sequences, and 8 sequences captured by drone simulators. Moreover, UAV123@10fps contains a total of 12 attributes, i.e., similar object (SOB), camera motion (CM), fast motion (FM), background clutter (BC), illumination variation (IV), out-of-view (OV), full occlusion (FOC), partial occlusion (POC), low resolution (LR), aspect ratio change (ARC) and viewpoint change (VC).

The DTB70 benchmark collects 70 UAV video sequences, including a total of 11 attributes, i.e., similar objects around (SOA), scale variation (SV), occlusion (OCC), fast camera motion (FCM), in-plane rotation (IPR), out-of-plane rotation (OPR), out-of-view (OV), background cluttered (BC), deformation (DEF), aspect ratio variation (ARV) and motion blur (MB).

The UAVDT benchmark includes a total of 50 video sequences for UAV object tracking, which has more than 37K image frames. This benchmark captures pictures from different weather conditions, flying altitude and camera view, which contains 9 attributes, i.e., a small object (SO), long-term tracking (LTT), camera motion (CM), object motion (OM), object blur (OB), illumination variation (IV), scale variation (SV), background clutter (BC) and large occlusion (LO).

Metric. As for the above-mentioned benchmarks, we adopt the most common metric, one-pass evaluation (OPE), to evaluate all trackers. This metric has the characteristic that the tracker with the initial position and scale information in the first frame needs to run through the entire video sequences and cannot be initialized during runtime. Besides, this metric includes two criteria, i.e., intersection-over-union (IOU) and center location error (CLE). The IOU is obtained by calculating the overlap ratio between the predicted box and the ground truth box. The CLE is gained by calculating the Euclidean distance between the center position of the predicted box and the ground truth. The unit of CLE is pixel. On the basis of the IOU and CLE, there are two evaluation criteria obtained by setting different thresholds, i.e., the success rate and precision rate. The IOU and CLE are formulated as follows, respectively:

(16) $$\begin{array}{cc} \; & \mathrm{IOU}=\frac{BB_{T}\cap BB_{G}}{BB_{T}\cup BB_{G}},\\ \; & \mathrm{CLE}=\sqrt{{\left(x_{a}-x_{b}\right)}^{2}+{\left(y_{a}-y_{b}\right)}^{2}}, \end{array}$$

where $BB_{T}$ and $BB_{G}$ represent the predicted box and the ground truth box, ∩ and ∪ denote the intersection area and union area operations of $BB_{T}$ and $BB_{G}$, $\left(x_{a},y_{a}\right)$ and $\left(x_{b},y_{b}\right)$ denote the center positions of $BB_{T}$ and $BB_{G}$.

The success rate plot shows the percentage of frames whose IOU is larger than a given overlap threshold varied from 0 to 1. The area under the curve (AUC) of each success rate plot ranks the tracking method when IOU is larger than the overlap threshold of 0.5, i.e., IOU ≥ 0.5. The precision rate plot illustrates the percentage of frames whose CLE is within the thresholds varied from 0 to 50 pixels. In addition, the distance precision (DP) represents the percentage of frames whose CLE is below a given threshold. When this given threshold is set to 20 pixels, the DP is used to rank the tracking method in the precision rate plot.

### 4.3. Comparison with Handcrafted-Based Features

#### 4.3.1. Analysis of Tracking Performance

**Figure 2:**
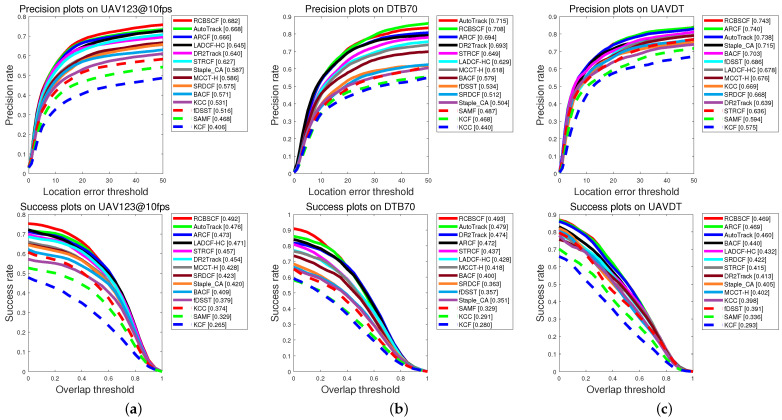
Precision and success plots of RCBSCF as well as other 13 state-of-the-art trackers on (**a**) UAV123@10fps, (**b**) DTB70 and (**c**) UAVDT benchmarks.

In this section, we have compared our tracker RCBSCF with other 13 state-of-the-art handcrafted features-based trackers. Experimental results are shown in Figure 2.

UAV123@10fps. Figure 2a illustrates the precision and success rates of all comparison trackers at the UAV123@10fps benchmark. Our tracker RCBSCF ranks first among all comparison trackers. In addition, the RCBSCF tracker has an improvement of 1.4–1.6% in precision rate and 1.6–1.9% in success rate against the state-of-the-art trackers (i.e., AutoTrack, ARCF). Compared with the baseline tracker SRDCF, our tracker RCBSCF outperforms by 10.7% in precision rate and 6.9% in success rate, respectively.

DTB70. In Figure 2b, our tracker RCBSCF does not perform as well as the AutoTrack tracker in precision rate and the precision rate of the RCBSCF tracker is lower than the AutoTrack tracker by 0.7%. However, in success rate, the proposed tracker is higher than the AutoTrack tracker by 1.4%. In addition, compared with the state-of-the-art trackers (i.e., ARCF, DR2Track). Our tracker raises the precision rate by 1.4–1.5% and the success rate by 2.1–1.9%, respectively. Compared with the baseline tracker SRDCF, the proposed tracker improves the precision and success rates by 19.6% and 13.0%, respectively.

UAVDT. In Figure 2c, our tracker achieves the best scores in precision and success rates. Although compared with the state-of-the-art trackers (i.e., AutoTrack, ARCF), the precision and success rates of our tracker RCBSCF are not much higher than theirs. The proposed tracker has a significant performance improvement over other trackers, such as LADCF-HC, DR2Track, and MCCT-H. Besides, compared with the baseline tracker SRDCF, our tracker RCBSCF improves the precision and success rates by 7.5–4.7%, respectively.

Overall performance. In Table 1, we have evaluated the overall performance of RCBSCF and other 13 state-of-the-art trackers using the average values of success and precision rates and the tracking speed (FPS) on all UAV benchmarks. By comparing the average success and precision rates of each tracker, the proposed tracker RCBSCF has the highest average success score (0.485) and achieves the best average precision score (0.711). In addition, Table 1 shows the tracking speed of each tracker. KCF achieves the top tracking speed (574.78 FPS) followed by fDSST (173.25 FPS) and Staple_CA (47.29 FPS). Although the tracking speeds of KCF, fDSST and Staple_CA are significantly faster than our tracker RCBSCF (36.21 FPS), their tracking performance of them is lower than our tracker in the average success and precision rates. Meanwhile, as can be seen in Table 1, our tracker RCBSCF has an important improvement by 8.2–12.3% in the average success and precision rates over the baseline tracker SRDCF. Furthermore, the proposed tracker can reach ∼36 FPS on a single CPU for online real-time UAV tracking.

#### 4.3.2. Attribute-Based Analysis

In order to fully verify the performance of our tracker RCBSCF in different UAV scenarios, we have conducted a comprehensive attribute analysis by using RCBSCF and other state-of-the-art trackers in UAV123@10fps, DTB70 and UAVDT benchmarks. Table 2 illustrates the success and precision rates of all trackers with handcrafted features in different attribute scenarios. It can be seen in Table 2 that our tracker RCBSCF ranks first on the comparative attributes in three UAV benchmarks. Simultaneously, to visualize the tracking results of RCBSCF, we have selected several challenging scenarios with different attributes for qualitative attribute-based analysis.

**Figure 3:**
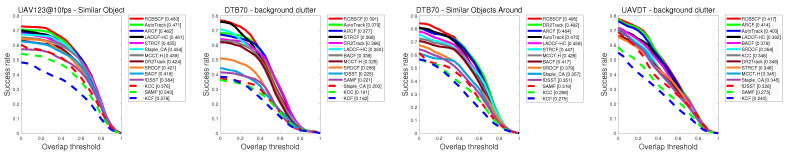
Success plots of our tracker RCBSCF and other 13 handcrafted features-based trackers in the scenarios of distractor attributes.

Distractor attributes. In all UAV benchmarks, the distractor attributes mainly include two challenge scenarios, i.e., similar object and background clutter. Figure 3 shows the evaluation results of RCBSCF and other top trackers (i.e., AutoTrack, ARCF, DR2Track, SRDCF) in distractor scenarios. The RCBSCF tracker achieves superior performance. In a scenario that includes a similar object, the distractor usually has a similar appearance to the target and is distributed in the background. Relying on the background-suppressed module, RCBSCF can learn the background information to suppress similar distractors in the tracking stage. In addition, in the circumstance of background clutter, the tracked target is surrounded by irrelevant objects in the background. With the response-consistent module, RCBSCF can maintain the change of the response map to be stable and repress the unexpected response peaks generated by irrelevant objects.

**Figure 4:**
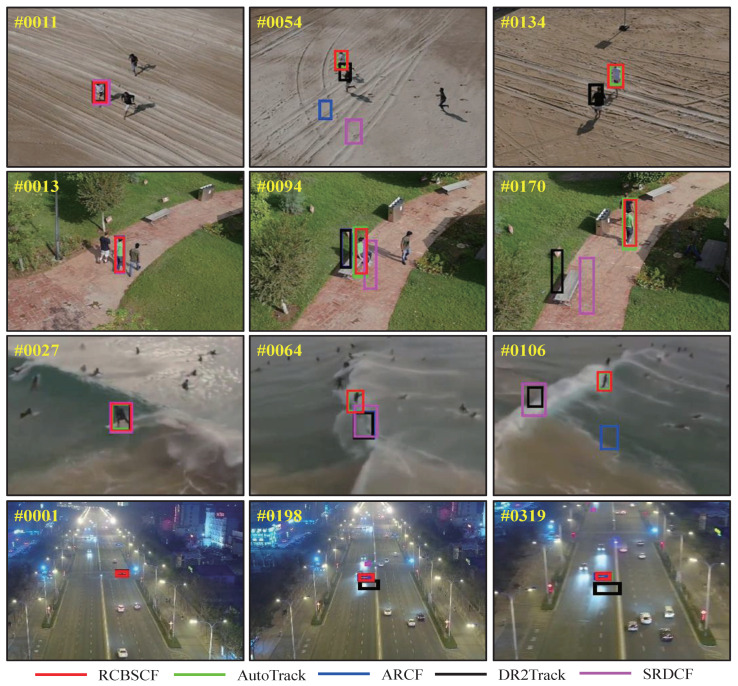
Qualitative analysis of our tracker RCBSCF and other top 4 handcrafted features-based trackers on four sequences that includes distractor attributes, i.e., group2_3_1 and group3_3_1 sequences in UAV123@10fps, surfing06 sequence in DTB70 and S0308 sequence in UAVDT.

In order to intuitively express the tracking performance of RCBSCF in the distractor scenarios, Figure 4 illustrates the tracking results in the sequences, i.e., group2_3_1 and group3_3_1 sequences in UAV123@10fps, surfing06 sequence in DTB70 and S0308 sequence in UAVDT from the top row to bottom row. In the group2_3_1 and group3_3_1 sequences, the tracked person is surrounded by other similar persons, which increases the difficulty of target tracking. Moreover, in the group3_3_1 sequence, the tracked person is affected by the green lawn in the background. Though the tracked person is in the two sequences that contain similar objects and background clutter, our tracker can always achieve stable tracking. Similarly, in the surfing06 sequence, the tracked person is affected by the clutters, which are irregular sea waves. In the S0308 sequence, the tracked car is disturbed by the light and other cars. Yet, our tracker RCBSCF can always track the target under the background interferences.

**Figure 5:**
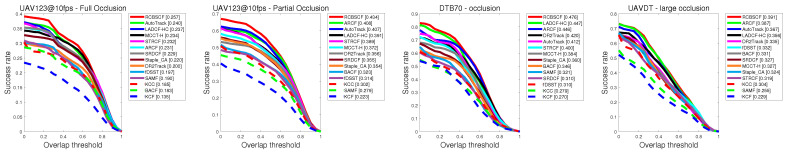
Success plots of our tracker RCBSCF and other 13 handcrafted features-based trackers in the scenarios of occlusion attribute.

**Figure 6:**
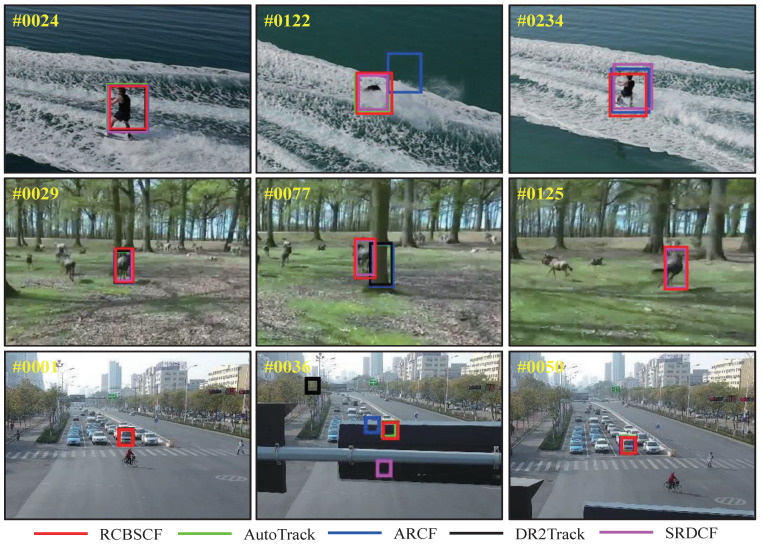
Qualitative analysis of our tracker RCBSCF and other top 4 handcrafted features-based trackers on three sequences that includes occlusion attribute, i.e., wakeboard5 sequence in UAV123@10fps, horse2_1 sequence in DTB70 and S0601 in UAVDT.

Occlusion attribute. UAV tracking usually encounters a situation where the target is partially or fully occluded. When the target is occluded, the appearance features will be polluted by the background information. Therefore, when the target returns to the view of the UAV, the UAV can not re-track it by using erroneous features. Here, we have evaluated the tracking performance of our tracker under the occlusion attribute on three UAV benchmarks. As shown in Figure 5, our tracker RCBSCF achieves the best success rate in four attributes, i.e., full and partial occlusion attributes in UAV123@10fps, occlusion attribute in DTB70 and large occlusion in UAVDT. Moreover, RCBSCF achieves a performance improvement of 16.6%, 2.8%, 7.9%, and 6.4% compared with the baseline tracker SRDCF, respectively. Figure 6 shows the qualitative comparison of tracking results in occlusion scenarios. From the top row to the bottom row, the image sequences are wakeboard5 sequence in UAV123@10fps, horse2_1 sequence in DTB70 and S0601 sequence in UAVDT. In the wakeboard5 sequence, although the tracked person is occluded by the sea waves, our tracker RCBSCF can still track the person reappearing in UAV view. In the horse2_1 sequence, the horse was once fully occluded by the stout tree. However, when the horse is no longer covered by the tree, our tracker RCBSCF can quickly locate the position of the horse and track steadily. In the S0601 sequence, the tracked car encounters the occlusion. Owing to the usage of our tracker, the car can be re-tracked when it reappears in UAV view.

**Figure 7:**
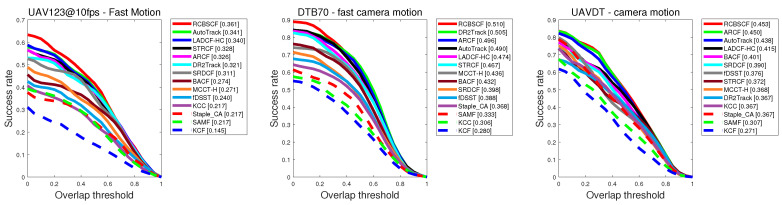
Success plots of our tracker RCBSCF and other 13 handcrafted features-based trackers in fast motion and camera motion scenarios.

**Figure 8:**
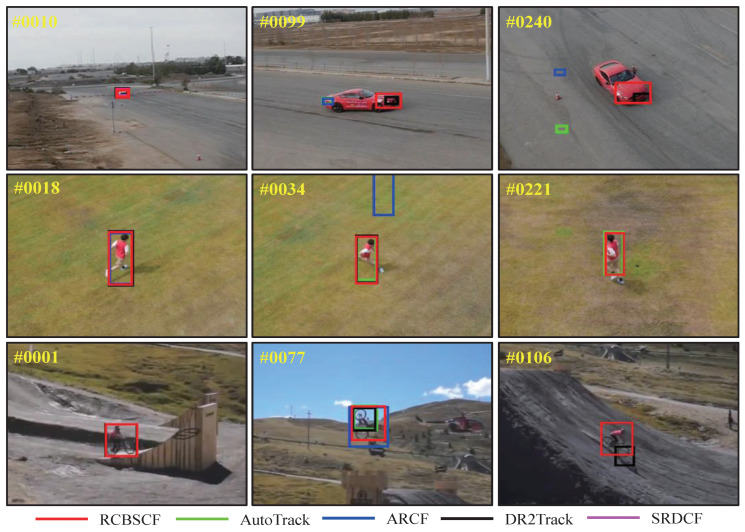
Qualitative analysis of our tracker RCBSCF and other top 4 handcrafted features-based trackers in the scenarios of other attributes, i.e., fast motion and camera motion. The sequences from top to bottom are car17_1 and person7_2_1 sequences in UAV123@10fps and MountainBike5 sequence in DTB70.

Other attributes. Apart from the analysis of the above attributes, we have intuitively shown the tracking results under other attributes scenarios, such as fast motion and camera motion, as illustrated in Figure 7. The first, second, and third rows in Figure 8 are car17_1 and person7_2_1 sequences in UAV123@10fps, MountainBike5 sequence in DTB70, respectively. In the car17_1 and person7_2_1 sequences, the tracked target is doing fast motion while the scale of its appearance changes. The RCBSCF tracker can achieve stable tracking. In the MountainBike5 sequence, the person is doing the fast in-plane rotation while the camera on UAV also moves for capturing the target. Our tracker RCBSCF is always able to track the biker successfully.

Attribute Analysis Result: The proposed tracker RCBSCF has an advantage in the scenarios with distractors, occlusion, and other situations such as fast motion and camera motion. In scenarios with distractors, trackers that encounter a similar object and cluttered backgrounds, such as ARCF, DR2Track and SRDCF, are prone to target drift. Especially, when the target has a similar color to the background or the target feature is not remarkable compared with the background, these trackers will incorrectly locate the target, but RCBSCF will still track the target stably. In scenarios with occlusion, when the target is partially occluded or temporarily fully occluded, trackers, e.g., AutoTrack and DR2Track, are easily affected by obstructions and lose the target. However, by avoiding the mutation of the filter and suppressing the background, RCBSCF can prevent the target from drifting in a large range and continue to track. In addition, when the target does fast motion with appearance scale changing or does in-plane rotation accompanying camera motion, trackers, e.g., AutoTrack, ARCF and SRDCF, cannot track the target stably compared with RCBSCF.

### 4.4. Comparison with Deep-Based Features

For a comprehensive evaluation of the proposed tracker, we have compared RCBSCF with other 9 state-of-the-art deep features-based trackers, i.e., ECO [31], SiamFC [44], IBCCF [45], UDT [46], UDT+ [46], TRACA [47], DSiam [48], LUDT [49], LUDT+ [49]. Table 3 illustrates the tracking performance by using the average precision and success rates and the average tracking speeds of all comparison trackers on three UAV benchmarks. As shown in Table 3, our tracker RCBSCF ranks second compared with other 9 deep features-based trackers and is 0.8% and 0.5% lower than the ECO tracker ranked first in success and precision rates. However, the tracking speed of our tracker RCBSCF has reached 36.21 FPS with CPUs, which is greater than the 11.80 FPS of the ECO tracker with GPUs. Besides, the tracking performances of the top three fastest trackers, namely TRACA, UDT and LUDT, are inferior to our tracker.

**Remark** **2.** *Compared with CPUs, deep features-based trackers utilize GPUs to complete convolution, pooling and other operations to improve the running speed. The proposed tracker RCBSCF mainly involves Fourier transform and Hadamard product operations without the requirement of GPUs. However, considering that the comparison of tracking speed usually needs to be conducted on the same platform, related operations in the ADMM iteration of RCBSCF are attempted to be completed by GPU acceleration in Matlab2019a with a single RTX2070 GPU. Finally, the tracking speed of RCBSCF with a GPU can reach 42 FPS, which is faster than the 36.21 FPS of RCBSCF with a CPU. Besides, although the speed (42 FPS) is not superior to TRACA, UDT and LUDT, it has surpassed other 6 deep features-based trackers and reaches the standard of real-time tracking (30 FPS).*

### 4.5. Ablation Study

In this section, we have conducted the ablation study of the proposed tracker on the UAV123@10fps benchmark for analyzing the effectiveness of each module. The RCBSCF-N represents the tracker without the response-consistent and background-suppressed modules. By introducing the response-consistent module into RCBSCF-N, we get the RCBSCF-RC tracker. In addition, the RCBSCF-BS is obtained by adding the background-suppressed module to RCBSCF-N. As shown in Table 4, the RCBSCF-RC has increased by 1.3% and 2.1% over the RCBSCF-N tracker in success and precision rates on UAV123@10fps benchmark, which proves the effectiveness of the response-consistent module. Simultaneously, compared to the RCBSCF-N tracker, the RCBSCF-BS also has increased by 2.2% and 3.7% in success and precision rates, which demonstrates that the background-suppressed module can benefit the improvement of the tracking performance. Furthermore, the RCBSCF is the proposed tracker equipped with both of the above modules and achieves the highest success and precision rates, i.e., 49.2% success rate and 68.2% precision rate. Therefore, the ablation study of the proposed tracker demonstrates that the response-consistent and background-suppressed modules can help to improve the success and precision rates and ameliorate the tracking performance.

### 4.6. Analysis of Key Parameters

**Figure 9:**
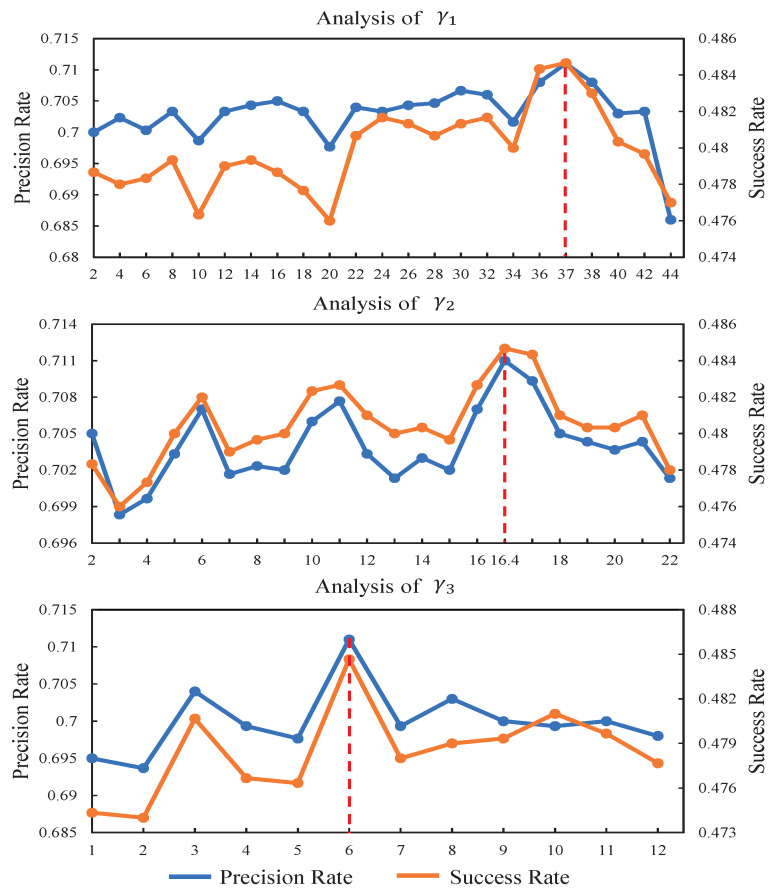
The average precision and success plots of our tracker RCBSCF on all UAV benchmarks by changing the values of $\gamma_{1}$, $\gamma_{2}$ and $\gamma_{3}$.

(1) $\gamma_{1}$ and $\gamma_{2}$ in the response-consistent module: Figure 9 reports the experimental results of the proposed tracker by changing the values of $\gamma_{1}$ and $\gamma_{2}$ when $\gamma_{3}$ is fixed. $\gamma_{1}$ varies from 2 to 44 with step size 2. $\gamma_{2}$ varies from 2 to 22 with step size 1. In this experiment, we first keep the value of $\gamma_{3}$ equal to 6 and fix its value. Then, we increase the value of $\gamma_{1}$ while $\gamma_{2}$ is set to 16.4. We obtain the best precision and success rates when $\gamma_{1}$ reaches 37. In addition, to analyze the impact of $\gamma_{2}$, we keep the values of $\gamma_{1}$ and $\gamma_{3}$ equal to 37 and 6, respectively. With the analysis of $\gamma_{2}$ in Figure 9, it can be seen that both the precision and success rates reach the top when $\gamma_{2}$ is set to 16.4.
(2) $\gamma_{3}$ in the background-suppressed module: In the experiment for studying the impact of $\gamma_{3}$, we change the value of $\gamma_{3}$ while $\gamma_{1}$ and $\gamma_{2}$ are all fixed. $\gamma_{3}$ varies from 1 to 12 with the step size 1. $\gamma_{1}$ and $\gamma_{2}$ are set to 37 and 16.4, respectively. With the analysis of $\gamma_{3}$ in Figure 9, it can be seen that when $\gamma_{3}$ is equal to 6, both the precision and success rates reach the best scores.

### 4.7. Failure Cases

**Figure 10:**
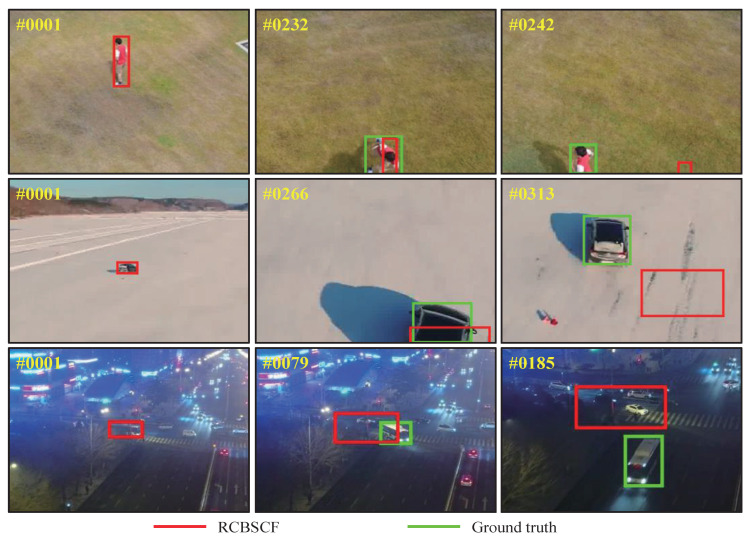
Failure cases of the proposed tracker. The sequences from the top row to the bottom row are person7_1_1 sequence in UAV123@10fps, Car6 sequence in DTB70 and S1401 sequence in UAVDT, respectively. The red box is depicted by the proposed tracker. The green box is depicted with the ground truth.

Figure 10 shows several failure cases of the proposed tracker on three UAV sequences, i.e., person7_1_1 sequence in UAV123@10fps, Car6 sequence in DTB70 and S1401 sequence in UAVDT from the top row to the bottom row. In the person7_1_1 and Car6 sequences, the tracked target encounters the scenarios with an out-of-view attribute. Since the tracked target is no longer in UAV view, the appearance model used for the training filter is completely polluted by the background information. When the target comes back into UAV view, the filter misguided by the background information can not identify and re-track the target, which causes the target loss. In the S1401 sequence, the target is in a scenario with poor lighting conditions and a cluttered background. The features extracted from the first frame can not effectively represent the target appearance. Therefore, when the appearance of the target changes during the following tracking process, the proposed tracker in this paper has been unable to track the target by means of the response-consistent and background-suppressed modules.

## 5. Conclusions

In this paper, we have proposed a response-consistent and background-suppressed correlation filter for real-time UAV tracking. First, a novel response-consistent module is constructed, which aims to keep the responses generated by the filter consistent with that of the previous frame. By using this module, the proposed tracker not only can avoid sudden changes in the target response caused by background interferences but also enables the learned filter to preserve the discriminative ability of the previous filter. Second, a novel background-suppressed module is developed, which makes the filter pay more attention to the background region with an attention mask matrix, and thus represses the response interferences of background distractors. Comprehensive experiments have demonstrated that the proposed tracker with the above two modules has achieved an excellent tracking performance compared with 22 other state-of-the-art trackers. Moreover, the tracking speed reaches 36 FPS, which is suitable for real-time UAV tracking.

However, the tracking performance (i.e., success and precision rates) of the proposed method has not reached an advanced level and there is much room for improvement. In this paper, the filter is trained with handcrafted features that have a limited expression on the appearance of the target compared with deep features. Thus, when encountering scenarios, e.g., appearance changes and more complex backgrounds, the proposed method is prone to target drift or loss. Besides, occlusion inevitably occurs during UAV tracking, which also leads to target loss. Since the proposed method does not equip with the target redetection mechanism, once the target reappears after occlusion, the target could not be captured with our method. In future work, we will improve the feature expression ability of the target by using lightweight deep features and plan to establish a redetection structure for addressing the occlusion problems.

## Figures and Tables

**Table 1:** The overall performance of our tracker RCBSCF and other state-of-the-art trackers, which is evaluated by average success and precision rates on all UAV benchmarks. The top three performances are highlighted by using red, green and blue fonts, respectively.

Trackers	Source	Avg. Success Rate	Avg. Precision Rate	Avg. FPS	CPU
**RCBSCF**	**This work**	0.485	0.711	36.21	*√*
DR2Track [35]	EAAI21	0.447	0.657	28.47	*√*
AutoTrack [10]	CVPR20	0.472	0.707	28.97	*√*
ARCF [13]	ICCV19	0.471	0.700	24.08	*√*
LADCF-HC [40]	TIP19	0.444	0.651	19.36	*√*
STRCF [8]	CVPR18	0.436	0.637	21.83	*√*
MCCT-H [41]	CVPR18	0.416	0.626	47.03	*√*
KCC [42]	AAAI18	0.354	0.546	28.9	*√*
Staple_CA [16]	CVPR17	0.388	0.596	47.29	*√*
BACF [12]	ICCV17	0.416	0.617	41.49	*√*
fDSST [43]	TPAMI16	0.376	0.579	173.25	*√*
SRDCF [6]	ICCV15	0.403	0.588	9.01	*√*
KCF [24]	TPAMI15	0.279	0.483	574.78	*√*
SAMF [27]	ECCV14	0.332	0.519	8.51	*√*

**Table 2:** The success rate of our tracker RCBSCF and other 13 state-of-the-art trackers in different attributes on UAV123@10fps, DTB70 and UAVDT benchmarks. The top three trackers are highlighted by using red, green and blue fonts, respectively.

Trackers	UAV123@10fps						DTB70				UAVDT		
	BC	SOB	CM	FM	FOC	POC	BC	SOA	OCC	FCM	BC	CM	LO
DR2Track [35]	0.267	0.424	0.413	0.321	0.200	0.356	0.366	0.492	0.420	0.505	0.348	0.367	0.335
AutoTrack [10]	0.313	0.471	0.460	0.341	0.240	0.407	0.379	0.470	0.412	0.490	0.403	0.438	0.367
ARCF [13]	0.291	0.462	0.435	0.326	0.231	0.408	0.377	0.484	0.446	0.496	0.414	0.450	0.387
LADCF-HC [40]	0.301	0.461	0.467	0.340	0.237	0.391	0.350	0.458	0.447	0.474	0.382	0.415	0.356
STRCF [8]	0.317	0.455	0.443	0.328	0.232	0.389	0.369	0.447	0.400	0.467	0.346	0.372	0.319
MCCT-H [41]	0.285	0.439	0.397	0.271	0.234	0.372	0.325	0.426	0.384	0.436	0.345	0.368	0.327
KCC [42]	0.237	0.376	0.331	0.217	0.185	0.302	0.191	0.298	0.279	0.306	0.348	0.367	0.304
Staple_CA [16]	0.297	0.454	0.384	0.217	0.220	0.354	0.200	0.357	0.360	0.368	0.345	0.367	0.324
BACF [12]	0.273	0.418	0.392	0.274	0.183	0.323	0.339	0.417	0.346	0.432	0.379	0.401	0.331
fDSST [43]	0.209	0.384	0.325	0.240	0.197	0.314	0.225	0.351	0.310	0.388	0.328	0.376	0.332
SRDCF [6]	0.263	0.421	0.399	0.311	0.229	0.355	0.256	0.379	0.310	0.398	0.358	0.390	0.327
KCF [24]	0.125	0.278	0.209	0.145	0.135	0.223	0.182	0.275	0.270	0.280	0.240	0.271	0.229
SAMF [27]	0.167	0.343	0.274	0.217	0.190	0.279	0.221	0.319	0.321	0.333	0.275	0.307	0.255
RCBSCF (Ours)	0.319	0.483	0.470	0.361	0.257	0.434	0.391	0.495	0.476	0.510	0.417	0.453	0.391

**Table 3:** The overall performance of our tracker RCBSCF and other 9 deep features-based trackers on all UAV benchmarks. The top three performances are highlighted by using red, green, and blue fonts, respectively.

Trackers	Source	Avg. Success Rate	Avg. Precision Rate	Avg. FPS	GPU
**RCBSCF**	**This work**	0.485	0.711	36.21	×
ECO [31]	CVPR17	0.493	0.716	11.80	*√*
SiamFC [44]	ECCV16	0.479	0.706	40.10	*√*
IBCCF [45]	ICCV17	0.445	0.648	2.40	*√*
DSiam [48]	ICCV17	0.468	0.696	17.82	*√*
TRACA [47]	CVPR18	0.400	0.594	77.83	*√*
UDT [46]	CVPR19	0.433	0.622	48.53	*√*
UDT+ [46]	CVPR19	0.454	0.683	34.83	*√*
LUDT [49]	IJCV21	0.416	0.593	45.27	*√*
LUDT+ [49]	IJCV21	0.448	0.681	33.69	*√*

**Table 4:** Ablation study of the proposed tracker on UAV123@10fps benchmark.

	RCBSCF-N	RCBSCF-RC	RCBSCF-BS	RCBSCF
Success rate	0.453	0.466	0.475	0.492
Precision rate	0.628	0.649	0.665	0.682

## Data Availability

Data are contained within the article.
